# On Medical Reform

**Published:** 1809-12

**Authors:** 


### *To the Editors of the Medical and Physical Journal.*

GENTLEMEN,

"Unde sit, ut ex quibusdam academiis redeant doctores parum docti, nihil minus quam apti ad medicinam, aut docendam, aut faciendam.

"Sicut igitur istas academiarum constitutiones non improbo, sed veneror; abusus tamen tam multos probare nemo potest."

BY the Circular Letter from Dr. Harrison, upon the subject of Medical Reform, which, together with the resolutions passed by the Medical Society at Horncastle, have been published in your Journal of this month, it appears that the measures projected by that gentleman have not, as was by many supposed, been entirely abandoned. As the question when it was first agitated was not very generally understood, and soon became confused and involved in the discussion of the rights and privileges of the several corporate bodies already in existence, I am sorry that the zealous promoters of it have not published a detail of the principal heads of the bill to be laid before parliament, and of the leading features of the plan, before they called upon their medical brethren for pecuniary aid. In the propriety of the object, I believe no man will differ from them—the exclusion of quacks and pretenders from the exercise of the profession, and the improvement of the regular race. But I apprehend that many will differ about the means of effecting so difficult a point, and all agree that a power so extensive in its operations should not be wantonly bestowed, or loosely defined.

By the way, I may observe, that it seems to be the fate of Dr. Harrison, like many other modern reformers, to be almost singular and unassisted in his exertions; and one would suppose that it did not argue well for the necessity or expediency of a measure which affects so large a body as the whole medical profession, regular and irregular, that it has been left to the entire guidance of a society little known beyond their own country. Indeed, it appears to us country practitioners, who are accustomed to look up to the "enlightened metropolis" for the origin of most of the improvements by which we are benefited, that we have some reason to doubt the efficacy of measures, in which they not only have no part, but about which they do not seem at all to interest themselves. This observation would fail of having any weight, if it were, as Dr. H. seems to infer, fer, that in the country only, irregularities occur; whereas it is a most notorious fact, that it is in London almost exclusively, that "the vilest empiric is as much at liberty to exercise the curative art, as the most skilful and regular practitioner;"* and when they do infest the country, it is well known that, excepting a few solitary and obsolete "water-doctors" and "leg-doctors," they emanate entirely from the metropolis. I cannot suppose that a man of liberal sentiment would include under that honourable appellation the present numerous, respectable, and well-informed and useful class of practitioners, the country surgeons, as Dr. H. seems to do, when he says, "quacks, aware of their ignorance and misdeeds, artfully conceal them, by compounding and dispensing their own drugs;" for it is certain that all country practitioners do mix and dispense their own medicines. If surgeon-apothecaries are not to be allowed to act as physicians in ordinary, the obvious consequence must be that the number of physicians must be multiplied, and a corresponding depreciation in the recompense of their labours must take place; and instead of a guinea or two as a common fee, the Doctor must content himself with receiving a dollar, or a half-crown, as they do upon most parts of the continent. This will ultimately be the case, granting that, according to the original plan, the present race of practitioners are not made amenable to an *ex post facto* law.

There is another, I think, obvious fault in the plan proposed, which is, to oblige apothecaries to submit to a five years apprenticeship, when it is well known that all the art and perfect dexterity in compounding medicines may be learnt by a young man of ordinary capacity in one, two, or at most three years, if he is intended for a mere apothecary; after which period, if bound for five years, he is losing his time, and the just reward of his services, or is exposed to all the temptations which belong to an idle life. I believe, by the bye, that most people will allow, that it would be beneficial to oblige regular physicians to serve for a certain time in the apothecary's shop.

There are many other points in Dr. H's letter, which require comment, but which I pass over for the present; (together with his hint of the murderous propensities of army and navy practitioners, from whose misdemeanours it would seem we have more to fear than from the power of

---

* See Circular Letter.

of the French, "when the prosperity of the empire can only be affected by want of numbers,") to make a short observation or two upon the utility of general restrictions and coercive measures.

In the first place, one is naturally led to consider whether the faults of which the reformers complain be real, or formidable, or whether they be growing evils. I think so far from the latter being the case, that we have fewer incompetent practitioners, and infinitely fewer quacks and empirics than formerly, and, in short, that the practice of medicine has kept pace with, and, in a great measure, gone beyond, the age in improvement. There is scarcely a village of a respectable size but can boast of a medical man who has spent one, two, or more seasons in the London schools, where, not more than half a century ago, the lives and limbs of the inhabitants, except of some of the most opulent, were trusted to village quacks, and empirics and old women, who although they had not the means of puffing themselves off, were quite as dangerous, and a thousand fold more numerous than the advertising quacks of the present day. The poorest individual will not now trust himself with any but the regular practitioner; and such is the general diffusion of knowledge, that it is become almost impossible for the ignorant man to escape detection. The numbers which flock to the best medical schools, and the increasing respectability of their external appearance, are convincing proofs of the improving state of the profession. It must be allowed that men of merit and real information are frequently superseded by men comparatively ignorant, who succeed in practice by dint of activity, and by vain assumption; but it is to be remembered, that these are not always the grossly ignorant, and that the same objection would occur in any state of the profession.

I believe, that the art is no where left to work its own way so much as in Great Britain, and no where is it exercised more extensively, more ably, and more to the emolument of its professors. Here it has escaped the shackles of universities and corporations, and has gained, I may say, a monopoly of talent unequalled in any part of the world. I attribute no sinister motives to Dr. Harrison and his coadjutors, but I fear that they will be instrumental in raising an aristocracy, which will be prejudicial to the general interests of the republic; and in putting the manacles of authority upon a liberal art, which disdains to be ranked as a trade amongst men.

There must of course be an exception with regard to the army army and navy; here the case is very different. To men in the military professions, there is no choice of medical attendance, and there are not time and opportunity for discrimination; the strictest attention therefore ought to be paid to the abilities and acquirements of those who are admitted to practice in these branches of the state. The case here, I say, is wholly different; and as to the profession in general, I would advise Dr. H. and his friends in the words of the French politician, upon a somewhat similar occasion, *laisser le faire*. I have visited the schools of London and of Edinburgh, and from what I have seen in them, and of the profession in general, I am convinced that competition is the best assurance to the progress of medical knowledge; and I should be sorry to see the art shackled by laws which cannot fail to produce confusion and dissension amongst its professors, and which the prejudices and attachments of men will, in a little time, render nugatory to all but those to whom they cannot but be hurtful.

The present indifference of medical men to the plan proposed, seems to argue against it; and I trust that a ready access to the ear of the Minister will not influence Dr. H. or any body of reformers, in bringing forward a measure of such importance prematurely, and without due consideration.

There seems to be a great rage for ruling every thing by the iron hand of the law.

I am, &c.

A Lover of the Profession, but an Enemy to the Quackery of Corporate Bodies.

*Oct.* 28, 1809.

